# Knowledge, attitude, and practice towards uterine cervical cancer screening in Egyptian females: a nationwide cross-sectional study

**DOI:** 10.1186/s12885-025-13530-4

**Published:** 2025-02-11

**Authors:** Mostafa Behery Behery, Ammar Ayman Bahbah, Mohamed Mohamed Shawqi, Yara Mohammed El-Said, Leenah Naser Sherif, Hager Abdelaziz Ataallah, Enas Sherif Adwy, Reem El-Sayed Ageez, Asmaa Helmi Abo Elwafa, Noor Maged Badrawy Ahmed, Reem Elsaadany, Naser Abd El-Bary

**Affiliations:** 1 Faculty of Medicine, Menoufia University, Menoufia, Egypt; 2 Student Research Program (SRP), Faculty of Medicine, Menoufia University, Menoufia, Egypt; 3 Department of Emergency Medicine, Faculty of Medicine, Benha University, Benha, Egypt; 4 Department of Emergency Medicine, Faculty of Medicine, Menoufia University, Menoufia, Egypt; 5 Faculty of Medicine, Alexandria University, Alexandria, Egypt; 6 Faculty of Medicine, Benha University, Benha, Egypt; 7 Faculty of Medicine, South Valley University, Qena, Egypt; 8 Faculty of Medicine, Ain Shams University, Cairo, Egypt; 9 Manchester Programme for Medical Education, Faculty of Medicine, Mansoura University, Mansoura, Egypt; 10 Clinical Oncology Department, Faculty of Medicine, Menoufia University, Menoufia, Egypt

**Keywords:** Cervical cancer, Screening, Knowledge, Practice, Egypt

## Abstract

**Background:**

Cervical cancer is a major cause of morbidity and mortality among women worldwide; it is ranked the 4th most common cancer among women globally. The current recommendation for cervical cancer (CC) screening involves the use of cytology examination methods like Pap smear. However, there is a lack of data on the practice of Pap smear screening in Egypt. Furthermore, understanding the knowledge, attitudes, and barriers related to cervical cancer screening among potential participants is crucial.

**Methods:**

In this cross-sectional study, we conducted interviews with female patients aged 21 years or more who visited outpatient clinics in six university hospitals across Egypt. The survey aimed to assess participants’ compliance with CC screening guidelines, their knowledge of and attitude toward CC screening, and their perception of potential barriers.

**Results:**

A total of 897 participants from the six study centers completed the survey. Only a small percentage (1.1%) of participants had undergone CC screening, although only (0.8%) of the participants were referred to do CC screening. The referral rate was more likely to be higher in participants who had one of their first-degree relatives or the surrounding people with a history of CC compared to those who have no one with CC (37.5% vs. 0.4%, *P* < 0.001; 5% vs. 0.6%, *P* = 0.035 respectively). Moreover, the referral rate was higher among participants who discussed CC and CC screening with their physicians (15.8% vs. 0.5%, *P* < 0.001; 23.5% vs. 0.3%, *P* < 0.001 respectively). Knowledge of CC screening was limited, with only 2.7% of respondents demonstrating good actual knowledge. However, after introducing the concept of CC screening to all participants, the majority (74.6%) showed a positive attitude towards undergoing the procedure. Lack of knowledge about the procedure, its tool, the place to do it, and financial burden were cited as the most common barriers to CC screening (79.8%, 65.9%, 64.2%, and 53.2%, respectively).

**Conclusion:**

Despite inadequate knowledge about CC screening, there is a positive attitude toward it among eligible participants in Egypt. This lack of knowledge likely contributes to low compliance with current CC screening guidelines and calls for national-level efforts to address this issue.

## Introduction

Cervical cancer (CC) is a major cause of morbidity and mortality among women worldwide. It is the 4th most common cancer among women globally, with an estimated 604,127 new cases and 341,831 deaths in 2020 [1–3]. African countries exhibit the highest mortality rates related to cervical cancer [2].

Regular screening helps in the early detection of cervical cancer and thus improves disease prognosis [4]. The United States Preventive Services Task Force (USPSTF) recommends screening for cervical cancer in women aged 21 to 65 years. Women aged 21 to 29 should have a Papanicolaou (Pap) smear every three years, while women aged 30 to 65 years should undergo either a Pap smear every three years or an HPV test every five years [5]. Therefore, females are recommended to discuss their individual risk factors and screening options with their healthcare provider [6]. The most known risk factors for developing cervical cancer include human papillomavirus (HPV) types (16, 18, 31, 33, and 45), smoking, and marriage before the age of 18 years [6]. The American Cancer Society recommends that girls get the HPV vaccine between the ages of 9 and 12 years, also teens and young adults through age 26 who are not already vaccinated should get the HPV vaccine as soon as possible. Moreover, teens who start the series late may need 3 shots instead of 2 [7].

In Egypt, cervical cancer is the 13th most prevalent cancer among women and it is the 9th most common cancer among women aged 15 to 44 years [8]. In 2020, it was estimated that there were 1,320 new cases of cervical cancer and 744 associated deaths in Egypt [3]. In 2002, an Egyptian national screening program was undertaken to assess the prevalence and risk factors associated with pre-invasive and invasive cervical cancer. The study included 5453 women aged 35 to 60 years and the prevalence of pre-invasive high grade lesions and invasive cervical cancer was 0.5% and 0.04%, respectively [9].

A recent study was conducted to assess the practice related to cervical cancer screening, and HPV vaccination among gynecologists and obstetricians in Egypt which concluded that most participants had poor knowledge and practice toward cervical cancer screening and HPV vaccination [10]. Currently, there is no structured nationwide screening program for cervical cancer in Egypt. Therefore, the screening totally depends on females’ perceptions and their healthcare provider’s recommendations. Studies have proved that Physician recommendation is a crucial factor in cancer screening performance and adherence [11–14]. Egypt has launched some ambitious initiatives to raise awareness about the early detection of cervical cancer in selected governorates [15]. However, the current knowledge, attitudes, and practice of Egyptian females toward cervical cancer screening on a national scope are still unknown. We therefore aim to explore the knowledge, attitude, practice, and barriers regarding cervical cancer screening among women in Egypt.

## Methods

We conducted a cross-sectional study to evaluate the knowledge, attitudes, and practices regarding uterine cervical cancer screening among Egyptian females. The study was approved by the Institutional Review Board of the Faculty of Medicine at Menoufia University, Egypt (7/2023ONCO1).

### Study Population and setting

The study included Egyptian females aged between 21 years or more who were attending outpatient clinics in selected university hospitals. Out of the 26 governmental university hospitals in four regions, we randomly selected six university hospitals using stratified random sampling based on geographical distribution to represent all regions. The selected university settings were Menoufia and Mansoura (representing the Delta Region), Ain Shams and Benha (representing the Cairo region), Alexandria (representing the North Coast region), and South Valley (representing the Upper Egypt Region). Females with a previous history of cervical cancer were excluded from the study.

### Questionnaire development

The survey was developed and validated by the study investigators after reviewing relevant literature. Pilot testing of the questionnaire was conducted on 10 patients from each center, with a total of 60 patients, to assess language comprehension, clarity, and the time needed to complete the questionnaire. Based on the pilot results, the questionnaire was modified accordingly. The responses from the pilot testing were excluded from the final analysis.

The survey consisted of six sections: sociodemographic data, relevant history, knowledge of cervical cancer screening, attitude toward cervical cancer screening, practice of cervical cancer screening, and barriers preventing cervical cancer screening. The history section included questions about previous cancers and gynecological diseases. We included an introductory statement before the knowledge section to differentiate between the uterus and cervix, specifically to clarify the distinction between uterine fibroids and cervical cancer. The knowledge section included a self-reported Knowledge scale of 10-points. Additionally, four knowledge questions were used to assess the actual knowledge, with a score of 3 or more out of 4 considered as good actual knowledge. Before the attitude section, an introduction to cervical cancer screening was provided. Similar to the knowledge section, a self-reported attitude scale of 10 points was used, with a score of 6 or more indicating a positive attitude. Participants also answered questions regarding barriers to undergoing cervical cancer screening and whether they had ever been recommended to have cervical cancer screening or receive the HPV vaccine.

### Data collection

A single investigator was recruited from each center to reach the target population in their respective university outpatient clinics. Data was collected through structured interviews conducted in the waiting rooms of the clinics. The investigators were trained to obtain verbal consent from participants after explaining the aim, objectives, and methodology of the study.

### Statistical analysis

Statistical analysis was performed using SPSS version 28. Descriptive data were reported using frequencies and percentages for categorical variables, as well as mean, median, standard deviation (SD), range, and interquartile range (IQR) for continuous variables. Pearson’s Chi-square and Fisher’s exact test were used to explore the statistical significance of categorical variables. All tests were two-sided, and a p-value less than 0.05 was considered significant.

The appropriate sample size was calculated using Raosoft.com based on a 95% confidence interval, a 3.5% margin of error, and a 50% response rate. The anticipated sample size was initially calculated to be 755 females.

## Results

### Results and characteristics of participants

We included 897 female patients from six different Egyptian university hospitals. The mean age of our participants was 40.32 ± 12.37 years. Most participants lived in rural areas (59.6%, *n* = 535), with a high educational level (60.4%, *n* = 544), married (78.9%, *n* = 708), unemployed (81%, *n* = 727), with low income (57.4%, *n* = 515) and self-funded medical care (79.0%, *n* = 709). (Table 1)

As regards medical history, 66%, 23.5%, and 25.3% of participants did not use contraceptive methods, participated in the breast cancer screening campaign conducted by the Ministry of Health during the last few years, and had a gynecological disease, respectively. Only 7.4% of participants reported having previous cancer other than cervical cancer. However, only 0.9% reported cervical cancer in their first-degree family members, and 4.5% reported cervical cancer in their surrounding people apart from the first degree relatives. Also, about 2% of participants discussed CC and CC screening with their physician. (Table 2)

**Table 1:** Sociodemographic of participants

Variables		
**Age**		**Mean** = 40.32 **SD** = 12.37
		**Number (%)**
**Residency**		
Rural		535 (59.6%)
Urban		362 (40.4%)
**Educational level**		
Low (Illiterate: preparatory school)		353 (39.4%)
High (Secondary school certificate or higher)		544 (60.6%)
**Marital status**		
Married		708 (78.9%)
Not Married		189 (21.1%)
**Work**		
Employed		170 (19.0%)
Unemployed		727 (81.0%)
**Income**		
High or Middle		382 (42.6%)
Low		515 (57.4%)
**Insurance**		
Covered by Government or employer		188 (21.0%)
Self-funded		709 (79.0%)

**Table 2:** Relevant medical history of the included participants

Variables	Number (%)
**Participants wsho use contraceptive method**	305 (34.0%)
**Participants who participated in breast cancer screening**	211 (23.5%)
**Participants who have been diagnosed with previous gynecological disease**	227 (25.3%)
**Participants who had cancer other than cervical cancer**	66 (7.4%)
**Participants who had a first-degree family member with cervical cancer**	8 (0.9%)
**Participants who had relatives or surrounding people apart from first-degree relatives with cervical cancer**	40 (4.5%)
**Participants who know anyone died from cervical cancer**	10 (1.1%)
**Participants who have discussed cervical cancer with their physician**	19 (2.1%)
**Participants who have discussed cervical cancer screening with their physician**	17 (1.9%)

### Knowledge

On a scale of ten, the median self-reported knowledge score was 1. Moreover, the actual knowledge score showed that 97.3% of the participants had poor actual knowledge. About 77.5% knew that CC screening should be done even without symptoms. Only 2.5%, 3%, and 13% knew the age, tools, and frequency of CC screening. (Table 3)

Good actual knowledge was more likely to be in participants with a high educational level (secondary school certificate or higher) compared with low educational level (4.4% vs. 0.00%, *p* < 0.001*), non-married compared to married participants (11.1% vs. 0.4%, *p* < 0.001), with high or middle income compared to low income (6.0% vs. 0.2%, *p* < 0.001), self-funded insurance compared to those with covered insurance (3.2% vs. 0.5%, *p* = 0.04). Moreover, actual good knowledge was more in participants that don’t use contraceptive methods (3.9% vs. 0.3%, *p* = 0.002), and discussed cervical cancer and cervical cancer screening with their physician (73.7% vs. 1.1%, *p* < 0.001* – 82.4% vs. 1.1%, *p* < 0.001*, respectively). (Table 4)

**Table 3:** Participants’ knowledge, attitude, and practice towards cervical cancer screening

Variables	Number (%)
**Knowledge**	
Self-perceived knowledge about CC screening (scale of 10)	**median** = 1 **IQR** = 0
Actual Knowledge about CC screening	
Good Knowledge (score ≥ 3)	24 (2.7%)
Poor Knowledge (score < 3)	873 (97.3%)
Participants who agreed that females should be screened even without symptoms	695 (77.5%)
Participants who knew the age of screening	22 (2.5%)
Participants who knew the tools of screening	27 (3.0%)
Participants who knew the frequency of screening	117 (13.0%)
**Attitude**	
Attitude towards CC screening (scale of 10)	**median** = 10 **IQR** = 5
Positive attitude (score ≥ 6)	669 (74.6%)
Negative attitude (score < 6)	228 (25.4%)
Participants who doubt the effect of CC screening in the early detection of CC	35 (3.9%)
Participants who will voluntarily intend to undergo CC screening	620 (69.1%)
Participants who will undergo CC screening if recommended by your physician	841 (93.8%)
Participants who will have a CC screening if there is a mass campaign about CC screening	744 (82.9%)
**Practice**	
Participants who underwent CC screening before	10 (1.1%)
Participants who have been recommended for CC screening	6 (0.8%)
Participants who have received HPV vaccine	0 (0.0%)

### Attitude

The median of self-reported score for the attitude of participants towards CC screening was 10 (IQR 5). Moreover, most participants (74.6%; *n* = 669) exhibited a positive attitude towards CC screening. Most participants didn’t doubt that CC screening can early detect CC (96.1%, *n* = 862), intend to undergo CC screening in the future (69.1%, *n* = 620), and about 83% will undergo CC screening if there is a mass campaign about it. Moreover, (93.8%, *n* = 841) will undergo CC screening if recommended by their physicians. (Table 3)

Positive attitude was more likely to be in participants with good actual knowledge compared with those with poor knowledge (100% vs. 73.9%, *p* = 0.001*), living in urban areas compared to rural areas (85.9% vs. 66.9%, *P* < 0.001), non-married compared to married (83.1% vs. 72.3%, *P* = 0.003), employed compared to unemployed (84.7% vs. 72.2%, *p* = 0.001), with high or middle income compared to low income (86.6% vs. 65.6%, *P* < 0.001), Furthermore, positive attitude was more likely to be in participants without contraceptive methods (77.2% vs. 69.5%, *P* = 0.012), with a previous history of cancer other than CC (92.4% vs. 73,2%, *P* < 0.001), and discussed CC and CC screening with their physician (100% vs. 74.0%, *P* = 0.006* – 100% vs. 74.1%, *P* = 0.010*, respectively). (Table 4)

**Table 4:** Association between knowledge and attitude with Sociodemographic and relevant medical history

	Knowledge			Attitude			
	Poor	Good	*P*. value	Negative	Positive	*P*. value	
**Actual knowledge level**							
● Good knowledge	-	-	-	0 (0.0%)	24 (100%)	0.001*	
● Poor knowledge	-	-	-	228 (26.1%)	645 (73.9%)		
**Residency**							
● Rural	518 (96.8%)	17 (3.2%)	0.257	177 (33.1%)	358 (66.9%)	< 0.001	
● Urban	355 (98.1%)	7 (1.9%)		51 (14.1%)	311 (85.9%)		
**Educational level**							
● High level (secondary school or higher)	520 (95.6%)	24 (4.4%)	< 0.001*	140 (25.9%)	403 (74.1%)	0.669	
● Low level	353 (100%)	0 (0.0%)		87 (24.6%)	266 (75.4%)		
**Marital status**							
● Married	705 (99.6%)	3 (0.4%)	< 0.001	196 (27.7%)	512 (72.3%)	0.003	
● Not Married	168 (88.9%)	21 (11.1%)		32 (16.9%)	157 (83.1%)		
**Work**							
● Employed	168 (98.8%)	2 (1.2%)	0.288*	26 (15.3%)	144 (84.7%)	0.001	
● Unemployed	705 (97.0%)	22 (3.0%)		202 (27.8%)	525 (72.2%)		
**Income**							
● High or Middle	395 (94.0%)	23 (6.0%)	< 0.001	51 (13.4%)	331 (86.6%)	< 0.001	
● Low	514 (99.8%)	1 (0.2%)		177 (34.4%)	338 (65.6%)		
**Health insurance**							
● Covered by Government or employer	187 (99.5%)	1 (0.5%)	0.04	41 (21.8%)	147 (78.2%)	0.201	
● Self-funded	686 (96.8%)	23 (3.2%)		187 (26.4%)	522 (73.6%)		
**Do you use any contraceptive methods?**							
● Yes	304 (99.7%)	1 (0.3%)	0.002	93 (30.5%)	212 (69.5%)	0.012	
● No	569 (96.1%)	23 (3.9%)		135 (22.8%)	457 (77.2%)		
**Have you participated in breast cancer screening?**							
● Yes	209 (99.1%)	2 (0.9%)	0.075	61 (28.9%)	150 (71.1%)	0.183	
● No	664 (96.8%)	22 (3.2%)		167 (24.3%)	519 (75.7%)		
**Do you have cancer other than cervical cancer?**							
● Yes	66 (100%)	0 (0.0%)	0.249*	5 (7.6%)	61 (92.4%)	< 0.001	
● No	807 (97.1%)	24 (2.9%)		223 (26.8%)	608 (73.2%)		
**Among your first-degree family members**,** has anyone had cervical cancer?**							
● Yes	8 (100%)	0 (0.0%)	1.000*	0 (0.0%)	8 (100%)	0.213*	
● No	865 (97.3%)	24 (2.7%)		228 (25.6%)	661 (74.4%)		
**Among your relatives and surrounding people apart from the first degree member**,** has anyone had cervical cancer?**							
● Yes	39 (97.5%)	1 (2.5%)	1.000*	15 (37.5%)	25 (62.5%)	0.073	
● No	834 (97.3%)	23 (2.7%)		213 (24.9%)	644 (75.1%)		
**Have you ever discussed cervical cancer with your physician?**							
● Yes	5 (26.3%)	14 (73.7%)	< 0.001*	0 (0.0%)	19 (100%)	0.006*	
● No	868 (98.9%)	10 (1.1%)		228 (26.0%)	650 (74.0%)		
**Have you ever discussed cervical cancer screening with your physician?**							
● Yes	3 (17.6%)	14 (82.4%)	< 0.001*	0 (0.0%)	17 (100%)	0.010*	
● No	870 (98.9%)	10 (1.1%)		228 (25.9%)	652 (74.1%)		

* Indicates the use of Fisher’s exact test

### Practice

Most participants had neither undergone CC screening before (98.9%, *n* = 887) nor were recommended for CC screening (99.2%, *n* = 891). Additionally, none of them had ever received the HPV vaccine before. The most important barrier that prevented participants from taking the HPV vaccine was the lack of knowledge about the existence of the HPV vaccine. (Table 3)

Regarding practice, the referral rate was more likely to be in participants living in urban areas (1.7% vs. 0.2%, *P* = 0.019*), had one of their first-degree relatives, or the surrounding people with a history of CC (37.5% vs. 0.4%, *P* < 0.001*; 5% vs. 0.6%, *P* = 0.035*, respectively). Moreover, the referral rate was higher among participants who discussed CC and CC screening with their physicians (15.8% vs. 0.5%, *P* < 0.001*; 23.5% vs. 0.3%, *P* < 0.001*, respectively). (Table 5)

**Table 5:** Factors associated with referral for cervical cancer screening

	Have you ever been recommended for CC screening?		
	No	Yes	*P*. value
**Residency**			
Rural	534 (99.8%)	1 (0.2%)	0.019*
Urban	356 (98.3%)	6 (1.7%)	
**Educational level**			
High level	539 (99.1%)	5 (0.9%)	0.710*
Low level	351 (99.4%)	2 (0.6%)	
**Marital status**			
Married	703 (99.3%)	5 (0.7%)	0.643*
Not married	187 (98.9%)	2 (1.1%)	
**Work**			
Employed	167 (98.2%)	3 (1.8%)	0.130*
Unemployed	723 (99.4%)	4 (0.6%)	
**Income**			
High or Middle	377 (98.7%)	5 (1.3%)	0.143*
Low	513 (99.6%)	2 (0.4%)	
**Health insurance**			
Covered by Government or employer	187 (99.5%)	1 (0.5%)	1.000*
Self-funded	703 (99.2%)	6 (0.8%)	
**Do you use any contraceptive methods?**			
Yes	304 (99.67%)	1 (0.33)	0.269
No	586 (98.99%)	6 (1.01%)	
**Have you participated in breast cancer screening?**			
Yes	209 (99.1%)	2 (0.9%)	0.670*
No	681 (99.3%)	5 (0.7%)	
**Do you have cancer other than cervical cancer?**			
Yes	66 (100%)	0 (0%)	1.000*
No	824 (99.2%)	7 (0.8%)	
**Among your first-degree family members**,** has anyone had cervical cancer?**			
Yes	5 (62.5%)	3 (37.5%)	< 0.001*
No	885 (99.6%)	4 (0.4%)	
**Among your relatives and surrounding people apart from the first-degree relatives**,** has anyone had cervical cancer?**			
Yes	38 (95%)	2 (5%)	0.035*
No	852 (99.4%)	5 (0.6%)	
**Have you ever discussed cervical cancer with your physician?**			
Yes	16 (84.2%)	3 (15.8%)	< 0.001*
No	874 (99.5%)	4 (0.5%)	
**Have you ever discussed cervical cancer screening with your physician?**			
Yes	13 (76.5%)	4 (23.5%)	< 0.001*
No	877 (99.7%)	3 (0.3%)	

*Indicates use of Fisher’s exact test

### Barriers

**Fig. 1:**
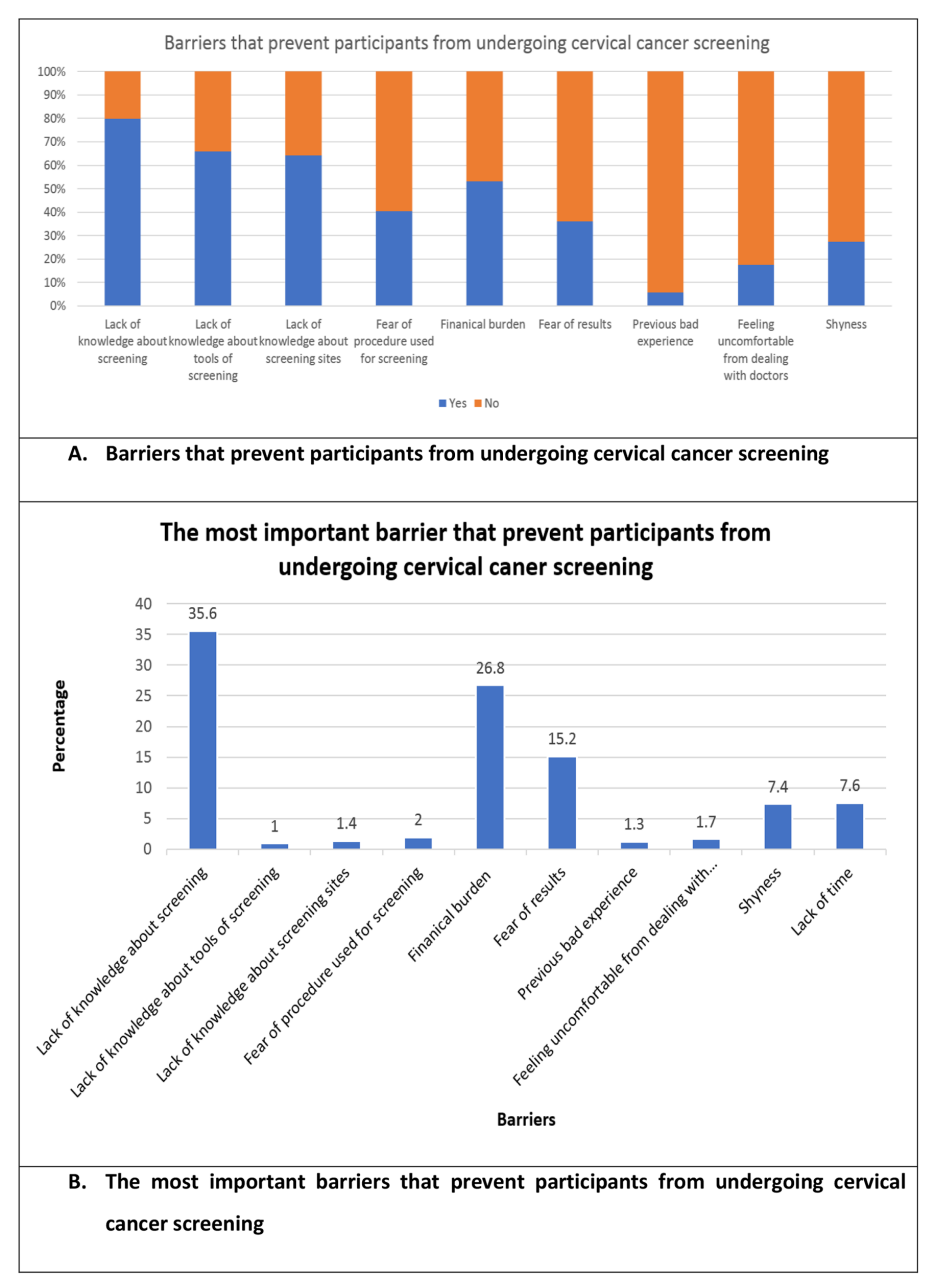
Barriers that prevent our participants from undergoing cervical cancer screening

Asking about the barriers that prevent our participants from undergoing CC screening, 79.8%, 65.9%, and 64.2% of the participants reported lack of knowledge about the procedure of CC screening, tools, and sites of screening as a barrier, respectively. Moreover, 53.2%, 40.5%, and 36% had a financial burden, fear from the procedure, and fear from the results, respectively. Also, Shyness was reported as a barrier among 27.4% of participants (Fig. 1A). By asking about the most important barrier that prevents participants from undergoing screening, lack of knowledge of CC screening (35.6%, *n* = 319), financial burden (26.8%, *n* = 240), and fear of results (15.2%, *n* = 136) were the top three factors (Fig. 1B).

Participants who chose lack of knowledge about CC screening were more likely to be married (81.5% vs. 73.5%, *P* = 0.016), low-income (85.6% vs. 72.0%, *P* < 0.001), live in rural areas (85.0% vs. 72.1%, *P* < 0.001), never shared in breast cancer screening (81.8% vs. 73.5%, *P* = 0.008), and never discussed CC (81.1% vs. 21.1%, *P* < 0.001*) and CC screening (81.1% vs. 11.8%, *P* < 0.001*) with their doctor. On the other hand, they were less likely to have a first-degree relative or know anyone apart from them with a previous history of CC (25.0% vs. 80.3%, *P* < 0.001*; 47.5% vs. 81.3, *P* < 0.001, respectively).

Regarding participants who chose financial burden as a barrier, they were more likely to have low income (60.8% vs. 42.9%, *P* < 0.001), with high educational level (61.5% vs. 47.8%, *P* < 0.001), unemployed (57.1% vs. 36.5%, *P* < 0.001), participated in breast cancer campaign conducted by ministry of health (65.4% vs. 49.4%, *P* < 0.001), and never discussed CC (53.8% vs. 26.3%, *P* = 0.018) or CC screening (53.8% vs. 23.5%, *P* = 0.013) with their physician.

Our participants who chose shyness as a barrier were more likely to be unmarried (33.9% vs. 25.7%, *P* = 0.026), with high level of education (32.9% vs. 19.0%, *P* < 0.001), live in rural (29.9% vs. 23.8%, *P* = 0.043), never participated in breast cancer screening (29.9% vs. 19.4%, *P* = 0.003), and discussed CC (52.6% vs. 26.9%, *P* = 0.013) and CC screening (52.9% vs. 26.9%, *P* = 0.026*) with their physician.

### Questionnaire impact

We assessed the influence of the questionnaire on participants’ attitudes towards undergoing CC screening. Therefore, we asked participants about their willingness to undergo CC screening both before and after completing the questionnaire. Initially, 69.1% were open to the idea of voluntarily undergoing CC screening. After the interview, the proportion increased to 80.4%, demonstrating the positive impact of the questionnaire.

Participants who were interested in CC screening were more likely to be employed (88.8% vs. 48.4%, *P* = 0.002), urban residents (87.3% vs. 75.7%, *p* < 0.001), married (82.2%vs. 73.5%, *p* = 0.008), those with previous gynecological history (93.4% vs. 76.0%, *p* < 0.001), and those with other cancers (90.9% vs. 79.5%, *p* = 0.025). Moreover, the increased interest in undergoing CC screening is more likely in females who never discussed CC (81.3% vs. 36.8%, *p* < 0.001*) and CC screening (81.4% vs. 29.4%, *p* < 0.001*) with their physician.

## Discussion

We conducted a multi-centric cross-sectional study to assess the current awareness of CC screening among females in Egypt. Our study reveals that the practice of CC screening in our sample has not met the desired levels despite the positive attitude. The level of knowledge about CC screening, which was also low, is considered a major factor that females do not practice screening.

Consistent with previous studies, the vast majority of participants (97.3%) had little to no knowledge about CC screening [16, 17]. In contrast, a study from the UAE found high levels of knowledge, which the authors attributed to the presence of a national CC screening program [18]. Interestingly, knowledge levels for breast cancer screening, which has been the focus of a national campaign in Egypt, were much higher compared to CC screening although most of the participants were not recommended to do mammograms [19]. Similarly, the knowledge of colorectal cancer screening, which lacks a national campaign, was also found to be low in Egypt [20]. The stark difference highlights the critical role of government-led initiatives and screening programs in improving public awareness.

Consistent with Baral et al. Most participants had a positive attitude towards CC screening despite their low knowledge [16]. Having previous cancer or other gynecological diseases was associated with a more positive attitude. Perhaps the previous experience made them realize how important early cancer diagnosis is. Also, we found that marital status affected the attitude as non-married females were more likely to have a positive attitude in contrast to other studies concluding that sexual activity is associated with a more positive attitude [21, 22]. Interestingly, 69.1% of participants expressed their intention to undergo screening, while 82.9% of them were willing to do screening if there was a mass campaign. It emphasizes the positive impact of national programs.

Similar to previous studies, we found that the practice of cc screening is very low as 98.9% of patients never went to cc screening [17, 23]. Lack of knowledge represents the top three barriers that prevented participants from undergoing cc screening. This finding was also reported in Asia, Ethiopia, and generally in middle and low-middle and low-income countries [24, 25].

Socioeconomic level affects the knowledge, attitude, and practice of cc screening. 53.2% of participants had a financial burden to undergo screening. Furthermore, low-income women were less likely to have actual knowledge or positive attitudes regarding cc screening. A similar finding was reported in another study which shows that women with enough family income were more likely to show intention to have a pap smear [26]. On a larger scale, Egypt is considered one of the lower-middle-income countries that are reported to have very limited coverage of CC screening [27].

We found that participants who had a discussion with their physicians about cervical cancer screening were more likely to have good knowledge and a positive attitude toward it. However, only 2% of participants reported having such a discussion, and even fewer were ever recommended for CC screening. Physicians need to consider counseling and recommending their patients to undergo screening. The effect of discussing cc screening on the intention to practice is evident as the ratio of participants willing to voluntarily undergo it increased from 69.1 to 80.4% just by the effect of our questionnaire.

Our study shows that cc screening is poorly covered mostly due to the ignorance of its presence. More efforts should be made to spread awareness about it such as national campaigns and screening programs provided by health facilities.

Our study had some limitations that should be taken into consideration. Firstly, we utilized a convenience sampling method to recruit participants from outpatient clinics at university hospitals, which may not be the most optimal approach. However, the large number of respondents in our study should enhance the generalizability of the results to some extent. Secondly, the analysis was constrained by the relatively small number of participants who were referred for cervical cancer (CC) screening. Therefore, caution should be exercised when interpreting associations with other parameters that were explored as secondary endpoints. Additionally, it is important to note that surveying the attitudes of participants who frequently visit healthcare facilities might be influenced by their health-seeking behavior. Furthermore, although we provided information about CC screening just before assessing participants’ attitudes, it is possible that residual knowledge gaps may have had some impact. Future studies could consider exploring participants’ awareness of CC symptoms and their potential associations with our study outcomes. This would provide further insights into the subject matter. Lastly, we did not investigate physicians’ perspectives on referral rates and potential barriers to CC screening. Exploring this aspect would be an area of interest for future studies and would complement the data we obtained from patients’ perspectives. Despite these limitations, it is worth noting that our study represents the largest assessment of cervical cancer screening practice in Egypt to date. This comprehensive analysis should provide valuable information to decision-makers, helping them identify and address potential challenges in the field.

## Conclusion

Despite the positive attitude, insufficient awareness regarding cervical cancer (CC) screening among eligible individuals in Egypt is evident, which likely contributes to low adherence to existing CC screening guidelines. The implementation of a well-structured nationwide screening program could serve as a pivotal strategy to enhance public knowledge and encourage widespread participation in CC screening within the community.

## Data Availability

No datasets were generated or analysed during the current study.
